# Dysregulation of PGC-1α-Dependent Transcriptional Programs in Neurological and Developmental Disorders: Therapeutic Challenges and Opportunities

**DOI:** 10.3390/cells10020352

**Published:** 2021-02-09

**Authors:** Laura J. McMeekin, Stephanie N. Fox, Stephanie M. Boas, Rita M. Cowell

**Affiliations:** 1 Department of Neuroscience, Life Sciences Division, Southern Research, Birmingham, AL 35205, USA; lmcmeekin@southernresearch.org; 2 Department of Cell, Developmental, and Integrative Biology, University of Alabama at Birmingham, Birmingham, AL 35294, USA; snfox@uab.edu (S.N.F.); boas2@uab.edu (S.M.B.)

**Keywords:** nuclear-encoded mitochondrial genes, nuclear respiratory factor 1, nuclear respiratory factor 2, mitochondrial transcription factor A, neuronal vulnerability, interneuron, spiny projection neuron, dopaminergic neuron, Parkinson’s Disease, Huntington’s Disease

## Abstract

Substantial evidence indicates that mitochondrial impairment contributes to neuronal dysfunction and vulnerability in disease states, leading investigators to propose that the enhancement of mitochondrial function should be considered a strategy for neuroprotection. However, multiple attempts to improve mitochondrial function have failed to impact disease progression, suggesting that the biology underlying the normal regulation of mitochondrial pathways in neurons, and its dysfunction in disease, is more complex than initially thought. Here, we present the proteins and associated pathways involved in the transcriptional regulation of nuclear-encoded genes for mitochondrial function, with a focus on the transcriptional coactivator peroxisome proliferator-activated receptor gamma coactivator-1alpha (PGC-1α). We highlight PGC-1α’s roles in neuronal and non-neuronal cell types and discuss evidence for the dysregulation of PGC-1α-dependent pathways in Huntington’s Disease, Parkinson’s Disease, and developmental disorders, emphasizing the relationship between disease-specific cellular vulnerability and cell-type-specific patterns of PGC-1α expression. Finally, we discuss the challenges inherent to therapeutic targeting of PGC-1α-related transcriptional programs, considering the roles for neuron-enriched transcriptional coactivators in co-regulating mitochondrial and synaptic genes. This information will provide novel insights into the unique aspects of transcriptional regulation of mitochondrial function in neurons and the opportunities for therapeutic targeting of transcriptional pathways for neuroprotection.

## 1. Introduction

Many studies implicate mitochondrial dysfunction as a key contributor to neuronal dysfunction in disease states. Mitochondrial dysfunction can arise by different routes, such as mutations in genes implicated in mitochondrial clearance and function, oxidative stress induced by environmental toxins, disruption of transcriptional programs for mitochondrial function, and reduction of cellular activity associated with hypoxia and/or hypometabolism. Here, we outline the biology of the regulation of nuclear-encoded mitochondrial genes in the brain, with a focus on the roles for the transcriptional coactivator peroxisome proliferator-activated receptor gamma coactivator 1-alpha (PGC-1α) in gene regulation. We present evidence for the disruption of PGC-1α-dependent gene programs in different brain disorders, including Huntington’s Disease (HD), Parkinson’s Disease (PD), autism, and schizophrenia. We go on to highlight the evidence for the neuroprotective potential of activating transcriptional programs for mitochondrial biogenesis, highlighting the potential neurotoxic effects of over-activating these pathways in vivo. We then evaluate the pharmacological strategies used previously to engage these transcriptional programs, pointing out the importance of considering neuroanatomical and cell-type-specific expression patterns of the intended molecular target when choosing the best candidates for target engagement studies. Altogether, this discussion should increase awareness of the complexities of mitochondrial gene regulation in the central nervous system and encourage the re-evaluation of strategies aimed at promoting mitochondrial health in neurons in disease states.

## 2. Mechanisms of Transcriptional Regulation of Nuclear-Encoded Mitochondrial Genes

The transcriptional regulation of nuclear-encoded mitochondrial genes follows the classic principles of regulation of gene transcription in eukaryotes [1], and this generic process has been reviewed previously in detail [2,3,4]. Briefly, consensus binding sequences in genomic DNA are bound by transcription factors and RNA polymerase II (pol II), initiating transcription [5,6,7,8]. Key transcription factors associated with the activation of genes involved in mitochondrial respiration are nuclear respiratory factor 1 (*Nrf1*) and nuclear respiratory factor 2 (NRF-2) subunits (encoded by *GABPA*, *GABPB1, GABPB2*), initially discovered to be regulators of cytochrome c (*CYCS*) and cytochrome oxidase subunit IV (*COXIV*), respectively [9,10,11]. Further studies indicate that these factors regulate programs for mitochondrial respiration and mitochondrial DNA replication by driving the expression of mitochondrial transcription factor A (*TFAM*; [12]). Their transcriptional activity can be amplified by the recruitment of transcriptional coactivators and chromatin modifiers [3,4,13], the majority of which do not directly bind DNA. Importantly, these coactivators and modifiers show cell-type-specific patterns of enrichment, enabling differential regulation of metabolic and mitochondrial genes by cell type and state.

The most well-characterized transcriptional coactivators of nuclear-encoded mitochondrial gene transcription are members of the peroxisome proliferator-activated receptor (PPAR) gamma coactivator-1 (PGC-1α) family and include PGC-1α, PGC-1β, and PGC-1α-related coactivator (PRC), encoded by the *PPARGC1A*, *PPARGC1B*, and *PPRC1* genes, respectively. These factors act downstream of intracellular signaling cascades to link extracellular stimuli to the transcription factors necessary for both basal and energy-demanding gene expression [14]. PGC-1α not only induces the expression of NRF-1 and NRF-2 subunits, but coactivates NRF-1 to drive the expression of genes for mitochondrial biogenesis and respiration [15]. In addition to NRF-1, PGC-1α can interact with a number of other factors, including members of the PPAR family (PPARα, PPARδ/β, PPARγ), members of the estrogen-related receptor family (ERRα, ERRβ, and ERRγ), initiator element binding factor (YY1), myocyte-specific enhancer factors (MEF-2A, MEF-2C, MEF-2D), forkhead box O1 (FOXO1), and others [1,16,17,18,19,20]. Additionally, PGC-1α associates with other cofactors including steroid receptor coactivator-1 (SRC-1) and CREB binding protein (CBP)/p300 [21] at its N-terminal activation domains [20]. SRC-1 and CBP/p300 possess inherent acetyltransferase activity, allowing them, through PGC-1α, to modify histones and recruit additional proteins to enhance transcription [22,23].

PGC-1α also interacts with PGC-1β, which is similarly expressed in tissues with high energy demands and regulates genes for oxidative phosphorylation, fatty-acid β-oxidation, and the citric acid cycle. While PGC-1α and PGC-1β can interact with a subset of the same transcription factors, there are those with which PGC-1β uniquely associates [19,24], such as SREBP1c in the liver, to trigger lipogenesis and triglyceride secretion [25]. The transcription factor interactions of PGC-1α and PGC-1β are reviewed extensively by Lin, Handschin, and Spiegelman [24]. However, less is known about PRC. While it can coactivate NRF-1, in contrast to PGC-1α, it is not preferentially enriched in energy-demanding tissues, is not associated with mitochondrial biogenesis in adipose tissue [26], and is expressed lowly in the brain (see below).

Repressors of PGC-1α have also been identified, including receptor-interacting protein 140 (encoded by *NRIP1*) [27], nuclear receptor co-repressor 1 (encoded by *NCOR1*), and silencing mediator of retinoic acid and thyroid hormone receptor (encoded by *Ncor2*) [16,28,29]. PGC-1α is also capable of enhancing gene transcription via displacement of repressor proteins at the promoter of its target genes [30,31], indicating there is a delicate balance between these sets of transcriptional regulators in fine-tuning the transcriptional needs of the cell. Interactions between corepressors and PGC-1α have been previously reviewed by Qi and Ding, 2012 [16].

While interactions with the PPARs, ERRs, and other nuclear receptor transcription factors are mediated by the Leu-Xaa-Xaa-Leu-Leu (LXXLL) motif in PGC-1α [19,24,32,33,34,35], the C-terminal portion of PGC-1α mediates interactions with the TRAP220/DRIP complex [36] and RNA splicing machinery. Two serine-arginine rich domains (RS) and a low specificity RNA recognition motif (RRM) mediate interactions with messenger RNA (mRNA) processing and splicing proteins [20,37,38]. Mutations in the RS and RRM domains impair the ability of PGC-1α to associate with RNA-processing factors and impair the ability of PGC-1α to induce downstream gene expression [37]. Though relatively unexplored, recent studies indicate that PGC-1α itself is capable of directly binding mRNA. For example, a recent study performed with primary hepatocytes demonstrated that PGC-1α binds directly to mRNA of proteins involved in glucagon and fasting responses, transcripts that are not direct transcriptional targets of PGC-1α, to influence gene expression and/or specify isoform specific expression [39]. Additional mechanisms by which PGC-1α can coordinate transcription and splicing are discussed in [20].

## 3. PGC-1α’s Roles in the Regulation of Gene Expression in Peripheral Tissues

PGC-1α was first discovered as an interacting factor of PPARγ and the thyroid hormone receptor in adipose tissue, with its expression causing an increase in mitochondrial mass and respiration and the up-regulation nuclear-encoded mitochondrial transcripts such as uncoupling protein 1 (*Ucp1*) [40]. Since then, expression of PGC-1α has been reported in tissues of high metabolic demand such as heart, liver, skeletal muscle, and brain. Though PGC-1α regulates overlapping metabolic genes in these tissues, it also regulates tissue-specific gene programs. Similar to its role in brown adipose tissue, PGC-1α regulates adaptive thermogenesis in skeletal muscle, through upregulation of *Ucp22* via NRFs [15]. In addition, PGC-1α expression is robustly induced following exercise in both rodents and humans [14,19,24], where it confers an oxidative phenotype to fast-twitch, glycolytic fiber-types [41] and enhances glucose as a source of ATP by regulating *Glut4* [42]. This utilization of glucose for ATP is shifted to fatty acid oxidation via the PGC-1α-ERRα-mediated regulation of pyruvate dehydrogenase kinase 4 (*Pdk4*), an inhibitor of pyruvate dehydrogenase activity [43], indicating that PGC-1α is capable of regulating the source of ATP to maintain glucose reserves under stressful conditions [19], depending on the type of interacting transcription factor present.

Mice lacking PGC-1α exhibit reductions in rearing and muscle strength as well as increased fatigability [44,45], likely due to the reduction in expression of genes involved in the citric acid cycle, electron transport chain, fatty acid oxidation and trafficking, and mitochondrial protein translation [46,47]. With regard to cardiac muscle, PGC-1α primarily mediates ATP production via fatty acid oxidation in addition to regulating mitochondrial biogenesis both in development and in response to short-term fasting [24,48]. In contrast to the reduction in mitochondrial number observed in skeletal muscle of PGC-1α null mice, no overt structural abnormalities or changes in number were documented in the mitochondria of PGC-1α null cardiac muscle [44,47]. However, transcripts involved in oxidative phosphorylation, fatty acid oxidation, and ATP synthesis were reduced [47]. These transcriptional changes translate to reduced contractility and heart rate as well as reduced responsiveness to beta-adrenergic stimulation [47].

A primary role of PGC-1α in the liver, and similarly seen in muscle, is to upregulate metabolic genes in response to fasting. PGC-1α expression is robustly increased during this state [14,24,49]. PGC-1α-dependent transcription leads to a metabolic shift from glucose utilization to glycogenolysis, gluconeogenesis, fatty acid oxidation, ketone body utilization and bile acid homeostasis. Many of these programs are regulated by the interaction of PGC-1α with a number of liver-enriched transcription factors [24]. Knockdown of PGC-1α in hepatocytes causes a reduction in genes involved in both fatty acid oxidation and gluconeogenesis [50,51]. While mitochondria in PGC-1α null hepatocytes exhibit no obvious changes in number or morphology, oxygen consumption is significantly reduced [46].

## 4. Cell-Type-Specific Roles for PGC-1α in the Brain

Interest in identifying PGC-1α’s potential roles in the brain stemmed from initial observations of brain vacuolizations in two lines of PGC-1α-deficient mice [44,46]. Mice with complete knockout of PGC-1α (PGC-1α null mice) show robust motor impairment, loss of neurofilament expression [46] and increased vulnerability to the toxic effects of 1-methyl-4-phenyl-1,2,3,6-tetrahydropyridine (MPTP) and kainic acid [52], while PGC-1α-deficient mice (with residual expression of an N-terminal truncation product [44]) show similar, although less severe, neuropathological and transcriptional changes. The motor impairment phenotype seen in the PGC-1α null line is recapitulated by conditional nervous system deletion using Nestin-cre-mediated deletion of *Ppargc1a* exons 3–5 [45]. As observed in peripheral tissues and cell types, PGC-1α overexpression increases mitochondrial density in neurons [53,54,55], enhances intrinsic mitochondrial function [56] and elevates ATP production [53] likely through the upregulation of *Tfam* and mitofusin 2 (*Mfn2*) [57] and other factors. PGC-1α is also capable of regulating glutathione peroxidase 1 (*Gpx1*) and superoxide dismutase 2 (*Sod2*) [52], the latter via the potential interaction with the reactive oxygen species (ROS)-sensitive transcriptional regulator, DJ-1 [58]. In line with these observations, reduction in PGC-1α expression caused by social isolation in mice are accompanied by an increase in markers of oxidative stress [59].

**Figure 1:**
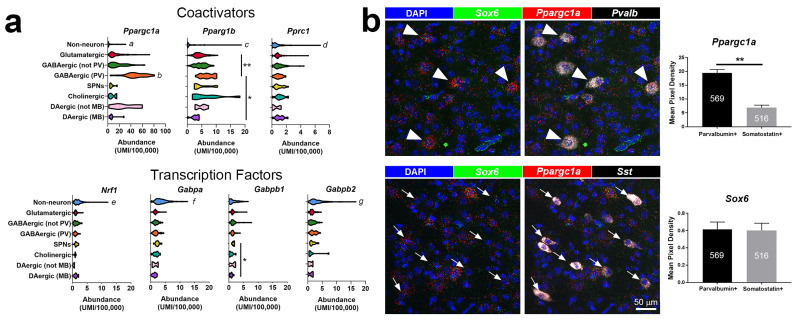
(**a**) A publicly available single-cell transcriptomic dataset [64] was used to explore cell-type-specific mRNA expression abundance in different neuronal populations of the mouse brain. Note the differences in the x-axes scales. (**b**) mRNA abundance for *Ppargc1a* and the interneuron-specific developmental marker *Sox6* was measured in neurons expressing parvalbumin (*Pvalb*, white arrowheads) or somatostatin (*Sst*, white arrows) mRNA using small molecule fluorescent in situ hybridization [62] in cortex from mice on postnatal day 14 (*n* = 3/group, total cell number indicated in white). Kruskal-Wallis analyses were used in (a), with * *p* < 0.05, ** *p* < 0.01, and letters indicating other statistically significant differences <0.05. *a* = different than all but cholinergic and DAergic (MB); *b* = different than all but cholinergic and DAergic (not MB); *c* = different than all but DAergic (MB); *d* = different than glutamatergic, GABAergic (not PV), and GABAergic (PV); *e* = different than all but SPNs, Cholinergic, and DAergic (MB); *f* = different than all but SPNs, Cholinergic, and DAergic (not MB); *g* = different than all but GABAergic (PV), SPNs, DAergic (not MB). Unpaired t-tests were used in (b); 165–208 cells and four representative sections /mouse, three mice; ** *p* < 0.01. UMI = unique molecular identifier. PV = parvalbumin-expressing neurons. SPNs = spiny projection neurons. MB = midbrain.

Despite the observation of robust motor impairment in PGC-1α null mice, initial studies revealed rather subtle gene expression changes in brain homogenates generated from the striatum [46,52]. These findings encouraged us to explore the cellular and regional distribution of PGC-1α expression in the brain. Using an antibody for PGC-1α [60], and, subsequently, small molecule fluorescent in situ hybridization [61,62], we observed the enrichment of PGC-1α expression in neurons which express glutamic acid decarboxylase 67 [60] and the calcium buffer parvalbumin (PV; [61]). Recently generated single-cell transcriptomics datasets [63,64] (Figure 1a) and additional studies with fluorescent in-situ hybridization (Figure 1b) have confirmed enrichment in PV-expressing interneurons, even as compared to other interneuron populations of similar developmental origins (*Sox6*/Somatostatin-positive interneurons; [65]; Figure 1b). In contrast, *Pparg1b* and *Pprc1* mRNA expression, while enriched in neurons with respect to non-neuronal populations, is lower in abundance throughout the brain. *Nrf1* and *Nrf2* subunit genes are expressed in both non-neuronal and neuronal populations (Figure 1a).

The observation of PGC-1α enrichment in PV-expressing neuronal populations is consistent with the high mitochondrial and cytochrome *c* content of PV-positive neurons as compared to other neuron-types [66]. Interestingly, most neurons expressing PV have very high firing rates [67] and elaborate axonal networks, with PV-positive interneurons (PV-INs) being responsible for the inhibition of large ensembles of pyramidal neurons in cortex [68]. Cortical PV-INs from PGC-1α null mice show a reduction in firing rate without an overt loss of PV-INs [69], while selective deletion of PGC-1α from PV-expressing cells causes impairments in memory and alterations in synchronous neurotransmitter release and region-specific circuit function [61,69,70,71,72]. Consistent with these observations, more recently, investigators have demonstrated that selective deletion of PGC-1α in GABAergic neurons using the *Dlx5/6*-cre line can cause habituation and sensorimotor deficits as well as hyperactivity [73].

Other cell types which express PGC-1α, although at a lower abundance, include glutamatergic neurons of the cortex and hippocampus [61], with higher expression in cortical layer V glutamatergic neurons [59]. Previous studies in mice have shown that deletion of PGC-1α from calcium/calmodulin-dependent protein kinase II alpha (*Camk2a*)-expressing neurons in the forebrain (glutamatergic neurons and striatal spiny projection neurons; SPNs) recapitulates the vacuoles seen in the PGC-1α null mouse [71]. Additionally, PGC-1α deletion in hippocampal glutamatergic neurons reduces mitochondrial density and dendritic spine density [53] and the expression of a number of genes involved in mitochondrial respiration and cytochrome *c* oxidase subunit I, suggesting a reduction in mitochondrial biogenesis and/or transcription [61]. Interestingly, deletion of PGC-1α from glutamatergic neurons is associated with more robust reductions in mitochondrial transcript expression in the hippocampus relative to neocortex [61]; functionally, these changes were associated with an increase in intrinsic excitability and enhanced axonal excitability in the temporoammonic pathway with minimal effects on the Schaffer collateral pathway [61]. Similar functional effects were observed in cortex, with a minimal effect on intrinsic excitability but an overall increased excitatory drive for both cortical and hippocampal postsynaptic targets [61].

Consistent with the relative deficiency of PGC-1α in striatal SPNs, we found relatively subtle transcriptional alterations when PGC-1α was depleted using the *Rgs9*-cre line [62]. With respect to dopaminergic neurons, which also express relatively low levels of PGC-1α, no loss of dopaminergic terminals are observed in PGC-1α whole body null mice [45]. However, a recent study demonstrated that viral-mediated knockdown of PGC-1α in dopaminergic neurons of aged mice causes cell loss [74], and a follow-up study reported a loss in dopaminergic neurons in the substantia nigra by 22 months of age with reductions in dopamine, DOPAC, and HVA in the striatum [75]. It is possible that the age-dependent reduction of PGC-1α, as has been suggested to occur during the process of neurodegeneration, could cause eventual cell loss.

It is important to note that, while mice lacking PGC-1α in the CNS [46] phenocopy the motor impairment observed in the whole body knockout, mice with deletion of PGC-1α from PV-expressing neurons do not show any motor impairment, despite the enrichment of PGC-1α in PV-expressing populations. In fact, Nestin (*Nes*)-cre-mediated deletion of PGC-1α is the only process which recapitulates the full motor phenotype of the PGC-1α null mouse [45], suggesting that disruption in PGC-1α-dependent pathways in multiple neuron types is required to generate the brain-knockout phenotype. It is important to note, however, that the PVcre line does not exhibit full recombination until 1–2 months of age [76,77]. Of note, whole body PGC-1α null mice exhibit a reduction in (PV-expressing) Purkinje cells and alterations in transcripts involved in neurotransmitter release and axonal integrity [78]. Thus, it is possible that the deletion of PGC-1α in PV-expressing cell types during development (especially PV-enriched brainstem and cerebellar neurons) could cause the motor impairment observed with brain-specific deletion.

## 5. Identification of Neuron-Enriched PGC-1α-dependent Transcripts Involved in Synaptic Function and Axonal Integrity

**Figure 2:**
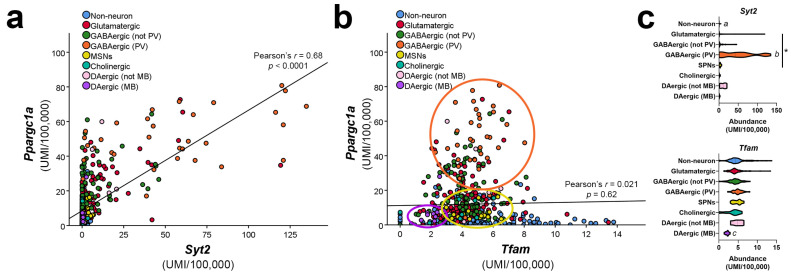
(**a**) *Ppargc1a* and synaptotagmin 2 *(Syt2)* or (**b**) *Ppargc1a* and *Tfam* mRNA expression is compared in different cell types using publicly available single-cell transcriptomic profiles [64]. Circles indicate clustering of datapoints for parvalbumin-positive interneurons (orange), spiny projection neurons (yellow), or dopaminergic neurons of the midbrain (purple). (**c**) Abundance of mRNA for *Syt2* and *Tfam* by cell type. Two-tailed correlation analyses were used in (a,b), and Kruskal-Wallis analysis was used in (c), with * *p* < 0.05, and letters indicating other statistically significant differences <0.05. *a* = different than all but cholinergic and DAergic (MB); *b* = different than all but DAergic (MB); *c* = different than all but cholinergic. UMI = unique molecular identifier. PV = parvalbumin-expressing neurons. SPNs = spiny projection neurons. MB = midbrain.

The observation of PGC-1α enrichment in interneurons led us to explore whether genes enriched in PV-INs are affected in whole body and cell-type-specific PGC-1α knockout mice. Transcriptional profiles of neuroblastoma cells overexpressing PGC-1α were mined for enrichment of interneuron-expressed genes [70], and subsets of these genes were found to be reduced in cortex of whole body and cell-type-specific PGC-1α null mice. Interestingly, the gene with the greatest upregulation with overexpression and reduction with knockout was *Pvalb*, the gene encoding PV, [70,79], without an overt loss in PV-INs [69]. Other genes included synaptotagmin 2 (*Syt2*; Figure 2a,c) and complexin 1 (*Cplx1*), genes involved in synchronous neurotransmitter release, and neurofilament heavy chain (*Nefh*), which was identified as a reduced gene in the original report of the PGC-1α null mouse [46], with the assumption that it was a marker of axonal loss. The confirmation of reductions in *Nefh* expression in striatum, cortex, and cerebellum of whole body PGC-1α null mice and mice with deficiency in pyramidal neurons [61] or SPNs [62] and the ability of PGC-1α to induce *Nefh* expression in vivo suggest that *Nefh* is a putative target of PGC-1α in multiple neuron types. Chromatin immunoprecipitation of PGC-1α from brain is required to determine whether these genes are direct targets of PGC-1α-containing regulatory complexes.

PGC-1α-dependent mitochondrial genes were also reduced in whole body PGC-1α-null mice, including isocitrate dehydrogenase 3a (*Idh3a*) and *Atp5a1* [61,70,78]. However, genes that are driven by PGC-1α in peripheral tissues such as *Tfam* are not reduced in expression in multiple brain regions of PGC-1α null mice, though *Tfam* is reduced in cardiac tissue from the same animals [79]. This suggests that while PGC-1α is necessary for some components of the mitochondrial respiratory chain and citric acid cycle, it is not required for maintenance of normal mitochondrial transcription in the brain. In fact, single-cell transcriptomic data indicate that *Tfam* is expressed ubiquitously in addition to *Nrf1*, raising the possibility that *Tfam* expression can be maintained by PGC-1α-independent transcriptional machinery. Interestingly, the lowest *Tfam* expression is observed in dopaminergic neurons of the midbrain relative to other cell types, including non-neurons (Figure 2b,c). This low expression level could be particularly relevant for understanding the biological contributors to dopaminergic neuron vulnerability to oxidative stress (discussed below).

Regarding PGC-1α’s potential neuron-specific roles, multiple studies show that NMDA receptor activation can increase PGC-1α expression [80,81,82] and that developmental deletion of *Grin1*, the obligatory subunit of the NMDA receptor, can reduce PGC-1α expression [59]. These findings are consistent with a potential role for PGC-1α in the developmental maturation of PV-INs, as PGC-1α-dependent genes *Pvalb*, *Syt2*, *Cplx1*, and *Nefh* are upregulated between postnatal day 7 and 14 in mice, when PV-INs show evidence for elaboration of processes and enhanced plasticity [83]. Enrichment of PGC-1α mRNA expression in PV-INs versus other interneurons can be observed as early as postnatal day 14, with enrichment versus other interneurons of similar neurodevelopmental lineage (*Sox6*/*Sst*-positive interneurons from the medial ganglionic eminence; Figure 1b). Modest increases in PGC-1α mRNA expression have also been observed in hippocampal neurons after exercise [84], status epilepticus [85], or after completion of memory retrieval tasks [86] with PGC-1α increasing the expression of brain-derived neurotrophic factor via upregulation of *Fndc5* (Irisin; [84]). PGC-1α expression can also be enhanced in striatal SPNs in response to cocaine exposure, with changes being selective for neurons expressing dopamine receptor *Drd1* [87]. Accumulating evidence also indicates the existence of brain-specific splice variants of PGC-1α [88] that are responsive to cellular stimuli, such as hypoxia [89], although the cell-type-specific distribution of these variants has not yet been determined. The cell-type-specific expression patterns of PGC-1α as well as its potential non-mitochondrial roles in neuron function are important to acknowledge when assessing therapeutic overexpression or targeting of PGC-1α for neuroprotection (see below).

## 6. PGC-1α and Transcriptional Dysregulation in Brain Diseases

### 6.1. Huntington’s Disease

The majority of studies investigating the roles for PGC-1α in neurodegeneration revolve around determining its roles as an etiological factor and/or potential therapeutic target in Huntington’s Disease (HD) and Parkinson’s Disease (PD; see list of studies summarized in Supplementary Table S1). HD is a severe progressive neurodegenerative disorder caused by a CAG repeat expansion in exon 1 of the huntingtin (*HTT*) gene [90]. Patients experience involuntary movements, abnormal gait and posture, and psychiatric symptoms, accompanied by cortical atrophy and the loss of SPNs of the caudate-putamen [91,92]. Mitochondrial dysfunction has been identified as a key contributor to HD pathology [93,94,95,96], with a reduction in mitochondrial complex activity being a reproducible finding in HD patient samples [97,98,99,100]. Murine models using quinolinic [101] or 3-nitropropionic acid [102] model striatal vulnerability and behavioral traits of HD by inducing excitotoxicity or directly targeting the mitochondria, respectively. Mutant huntingtin (mtHTT) itself can interfere with mitochondrial respiration, calcium buffering capacity, and ATP production [103,104]; array studies across multiple HD models have repeatedly identified a reduction in mitochondrial and synaptic transcripts in the striatum [105,106].

Various mechanisms of transcriptional dysregulation have been reported in HD [107,108,109,110,111], with studies indicating the disruption of PGC-1α-dependent and PGC-1α-independent transcriptional programs. Initial interest in a link between PGC-1α dysfunction and HD etiology was stimulated by the observation of ambulatory hyperactivity and striatal vacuolizations in the PGC-1α null mouse and reduced viability of SPNs cultured from these mice [46]. Follow-up studies found reductions in *PPARGC1A* mRNA expression in HD patient brains and cell culture and mouse models of HD [112,113,114,115,116,117,118] and suggested that mtHTT interferes with CREB-mediated transcription of *Ppargc1a* [112]. Importantly, these studies demonstrated that PGC-1α overexpression attenuates mtHtt-induced cellular toxicity [112,113]. Around the same time, another study documented a role for CREB in the regulation of PGC-1α in neurons in response to oxidative stress [52]. The inhibition of PGC-1α expression by mtHTT has been proposed to contribute to NMDAR-mediated excitotoxicity [117]. Interestingly, multiple studies documented disruption in PGC-1α-dependent transcriptional pathways in peripheral tissues as well, including brown adipose tissue [113], muscle [119], and liver [120]; this suggests the ability of mtHTT to interfere with PGC-1α-dependent transcriptional programs in different cellular contexts.

Subsequently, several studies found an association between variation the *PPARGC1A* locus and age of onset in HD [121,122,123,124], with the age-of-onset-associated haplotype encompassing the promoter of brain-specific isoforms [88,125]. However, it is important to point out that an association between age-of-onset and the *rs7665116* SNP in *PPARGC1A* was not replicated when phenotypic and genotypic stratification was taken into account [126]. Of note, *NRF1* and *TFAM* are genetic modifiers of HD [127], suggesting convergence of genetic risk on gene programs for mitochondrial function.

In addition to evidence for the disruption of PGC-1α expression and/or activity in HD, there is abundant evidence for the direct interaction of mtHTT with transcription factors [128,129,130], causing a disruption in gene expression and compromising cellular viability [108,131,132]. For example, mtHTT can prevent the transcriptional activator SP1 from binding to DNA to elicit transcription [133,134,135] (reviewed in [131]). SP1 can serve as both a mediator of PGC-1α interactions with other transcription factors and as a regulator of PGC-1α transcription itself [136]. mtHTT is also capable of interacting with CBP to reduce its expression [137,138,139,140] and with CREB [141], interfering in its interactions with TAFII130 and the activation of downstream genes [142], including PGC-1α.

mtHTT can also interact with p53 [137,143], a robust transcriptional regulator of multiple mitochondrial programs in neurons [144], increasing its expression and leading to mitochondrial dysfunction and reduced cell viability [143]. Absence of p53 ameliorates these deficits and prevents the onset of behaviors seen in HD models [143]. PGC-1α is reported to bind to and modulate p53 to promote cell survival, metabolic, and antioxidant transcriptional programs [145,146]. Heat shock transcription factor HSF1 could also be involved, as its binding at the PGC-1α promoter is significantly reduced in a cell culture model of HD and silencing of HSF1 in this model exacerbates cell death [127].

These studies led us to test whether a reduction in PGC-1α in SPNs is sufficient to cause transcriptional changes, ambulatory hyperactivity, and SPN loss. While we found that deletion of PGC-1α specifically in SPNs causes age-related ambulatory hypoactivity and a reduction in previously identified PGC-1α-dependent genes such as *Nefh* and *Idh3a*, it does not cause striatal atrophy [62]. Further, the expression of PGC-1α mRNA, as well as many of its dependent genes identified in other cell-types, was unchanged in the striatum of the HDQ [62] and R6/2 [45] mouse models. Considering the observations of changes in other models, it is possible that assessment of cell-type-specific transcriptional profiles (*Drd1* versus *Drd2*-expressing SPNs) may reveal changes consistent with past studies. Overall, though, these findings suggest that disruptions in PGC-1α expression and/or activity are not sufficient to cause an HD-like phenotype in mice, in the absence of mtHTT. Considering that the expression of mtHTT selectively in striatal SPNs fails to recapitulate motor deficits seen in animals with global expression [147], it is possible that PGC-1α deficiency is necessary in other cell types (striatal interneurons or cortical-striatal projection neurons) to generate an HD-like phenotype.

In fact, directed expression of mtHTT specifically in PV-expressing neurons causes ambulatory hyperactivity and cortical excitability [148]. Being that PV-expressing neurons depend heavily on PGC-1α, this model in particular may be best to understand not only how the circuit is disrupted in HD but the role of PGC-1α in regulating it. Aside from the more obvious cortico-striatal pathway affected in HD, it would be remiss to exclude the possibility that a reduction in PGC-1α in the cerebellum could impact disease progression. Several studies in HD models indicate a disruption in Purkinje cell firing rate and eventual cell loss as well as reductions in *Pvalb* mRNA expression [149,150]. In fact, Purkinje cells in the PGC-1α null mouse exhibit a reduction in firing rate and reductions in metabolic and synaptic transcripts and eventual cell loss [77], indicating an eventual need for studies of this transcriptional pathway in models of HD.

A compelling demonstration of the global downregulation of genes involved in oxidative phosphorylation in SPNs in human HD and mouse HD models was recently published [151]. In this study, the authors used single-nucleus RNA-sequencing and translating ribosome affinity purification to transcriptionally profile different neuron and glial types from human postmortem HD brain and HD mouse models. While genes involved in oxidative phosphorylation were reduced in both DRD1/*Drd1* and DRD2/*Drd2*-expressing SPNs, the latter were more robustly affected in mouse models. While no changes in PGC-1α expression were reported, the authors speculate that disruption of RARβ (*Rarb*), which is highly expressed in SPNs, could be involved [152]. Of note, PGC-1α can interact with members of the retinoid receptor family [153], so it is possible that some of the neuroprotective effects of PGC-1α overexpression in the striatum could be mediated through RARβ activation. It is also possible that splice variants of PGC-1α are decreased in these models; studies in both human postmortem tissue and HD models have reported a decrease in the N-terminal variant of PGC-1α with no effect on the full-length protein [118]. Further studies of the cell-type-specific regulation of these variants and the mechanisms underlying their regulation are warranted, particularly in disease settings.

How these transcriptional changes could give rise to a relatively selective loss of SPNs is unclear. A combination of cell- and non-cell-autonomous mechanisms are proposed to contribute to SPN vulnerability in HD (reviewed in [154]), some of which are related to cellular identity [155]. SPNs are characterized by significantly long projections that may render them more vulnerable to disruptions in mitochondrial trafficking and clearance. Further, striatal mitochondria, relative to those in other cell types, are particularly sensitive to fluctuations in calcium concentrations and, as a result, produce lower levels of ATP than other neuron types [156]. Additional potential reasons for SPN vulnerability include the interplay between GABA metabolism and the citric acid cycle; brain mitochondria have been shown to have lower content of succinate dehydrogenase, which could favor the maintenance of GABA levels over the conversion of succinate to fumarate [156,157,158]. However, not all GABAergic populations are vulnerable; striatal PV-INs are lost with advancing disease in HD patients [159] and in the R6/2 model [160] while cholinergic interneurons and interneurons expressing somatostatin and/or neuronal nitric oxide synthase are relatively preserved [161,162,163,164].

Considering the evidence for disruption in multiple transcriptional pathways in HD patients and cell and animal models, it is likely that any intervention will need to engage multiple targets. While recent efforts to knock down the expression of the *HTT* gene itself with antisense oligonucleotide therapy have shown promise [165,166,167], approaches are still needed to complement this strategy (see therapeutic strategy section, below).

### 6.2. Parkinson’s Disease

In contrast to HD which is an autosomal dominant disorder with high penetrance, PD is thought to arise from a contribution of genetic and environmental factors. Recent estimates of heritability in PD range from 16–36% [168], with the majority of mechanistic studies focusing on understanding molecular and cellular functions of rare genetic variants. Key clinical features of the disease include resting tremor, rigidity, and akinesia. Motor symptoms are accompanied by a progressive loss of dopaminergic neurons of the substantia nigra pars compacta (SNc) and, typically, the abnormal aggregation of the synaptic protein α-synuclein in deposits called Lewy bodies and Lewy neurites [169,170,171].

Although the exact mechanisms of dopaminergic cell loss are unclear, abundant evidence suggests the involvement of mitochondrial dysfunction [172,173,174]. Dopaminergic neurons of the SNc are especially vulnerable to mitochondrial toxins such as rotenone [173], paraquat [175], 6-hydroxydopamine [176] and MPTP [177,178,179]. A number of genes associated with autosomal dominant forms of PD including leucine-rich repeat kinase 2 (*LRRK2*), vacuolar protein sorting-associated protein (*VPS35*), and *SNCA* have been linked to mitochondrial dysfunction. Additionally, three genes associated with autosomal recessive forms of parkinsonism, *PARKIN (PARK2)*, *PINK1*, and *DJ-1 (PARK7)*, have roles in mitochondrial clearance in response to mitochondrial depolarization and oxidative stress (reviewed in [180]). The strongest evidence for a mechanistic link between PD and the dysregulation of transcriptional programs for nuclear-encoded mitochondrial genes stems from two lines of investigation: identification of the mechanisms underlying neuronal vulnerability with PARKIN loss-of-function [181,182,183,184,185] and the transcriptional profiling of dopaminergic neurons from postmortem tissue of patients with Lewy pathology [186].

The protein α-synuclein, encoded by the PD-linked gene, *SNCA*, is a major component of Lewy bodies which have recently been shown to also contain mitochondria, along with other organelles [187]. Point mutations (A53T and A30P) in the *SNCA* gene (*PARK1* locus) and triplication of the *SNCA* locus are associated with upregulation and subsequent increase in α -synuclein expression [188,189,190]. Studies from human iPSCs with A53T *SNCA* mutations identify a MEF2C/PGC-1α transcriptional pathway contributing to neuronal damage. In these neurons, MEF2C and PGC-1α were downregulated through oxidative stress that inhibits MEF2C’s ability to regulate PGC-1α which prevents its neuroprotective effects [191]. Similarly, in animals harboring the A30P mutation, PGC-1α was found to be downregulated, interfering with the transcription of neuroprotective genes [192].

LRRK2 mutations cause a form of PD which is clinically indistinguishable from idiopathic PD [193]. The most frequent mutation of LRRK2 (G2019S) causes an increase in its kinase activity, but it is unclear how this leads to PD. LRRK2 has been shown to be linked to mitochondrial dysfunction through regulation of mitochondrial motility; it has been shown to work in concert with PARKIN and PINK1 to modulate mitophagy [194,195]. Studies indicate that PGC-1α and the NAD-dependent protein deacetylase SIRT1 play a central role in cell metabolism and mitochondrial biogenesis [196,197]; deacetylation of PGC-1α by SIRT1 causes its activation and the transcription of genes involved in antioxidant defense [198,199,200]. Interestingly, iPSC-derived dopaminergic neuron cultures, as compared to glutamatergic cultures from LRRK2 G2019S patients, have diminished expression of the active PGC-1α and a subsequent increase in ROS [201]. In fact, a small-molecule activator of PGC-1α enhanced resistance against oxidative stress in human dopaminergic neurons [202].

Mutations in the *PARKIN* gene (~130 different mutations documented in ~1000 patients) cause early-onset PD with a median age of onset at 31 years of age [203,204]. PARKIN is an E3 ubiquitin ligase that plays a role in regulation of mitochondrial quality control via ubiquitination of toxic substrates for degradation by the proteasome [205,206]. Thus, PARKIN deficiency caused by loss-of-function mutations causes the accumulation of noxious substrates, leading to cellular stress [207]. One of these substrates is PARKIN-interacting substrate (PARIS/*ZNF746*/*Zfp746*), which accumulates in the absence of PARKIN expression. The accumulation of PARIS interferes with the expression of PGC-1α and NRF-1 via an insulin response element in the *Ppargc1a* promoter [181], mimicking reductions in *PPARGC1A* and *NRF1* mRNA expression in postmortem SNc from PD patients. Follow-up studies confirmed these findings in drosophila [183] and stem cell models [185], with the observation that PARIS-mediated dopaminergic vulnerability can also be rescued by overexpression of PINK1 [183].

Transcriptional profiling studies support the idea that PGC-1α-dependent pathways of nuclear-encoded mitochondrial genes are impaired in PD [186]. Laser capture microdissection of dopaminergic neurons from postmortem tissue of patients with Lewy pathology revealed a reduction in a number of PGC-1α-responsive genes, especially genes encoding proteins for respiratory complexes of the electron transport chain. While this study did not report reduction in *PPARGC1A* mRNA or PGC-1α protein, these data suggest the disruption of a transcriptional program for oxidative phosphorylation in these neurons in PD, potentially prior to cell loss.

Consistent with these data, a reduction in PGC-1α expression in multiple brain regions, including the caudate-putamen, is observed in both patients with advanced stage PD and animal models of PD [181,192]. Interestingly, *PPARGC1A* polymorphisms have been reported to influence the age of PD onset [208]. Two studies have indicated that full-length brain-specific splice variants of PGC-1α are reduced in postmortem SNc of PD patients [74,209]. In one of the studies, this reduction was accompanied by an increase in splice variants encoding truncated isoforms which can interfere with the activity of the full length variant [209]. Additionally, a loss of PGC-1α significantly exacerbates cell death in MPTP [52,210,211,212,213,214] and α-synuclein-induced cell death [192,215,216] models of PD, while overexpression of PGC-1α enhances autophagy and reduces rotenone- and α-synuclein-mediated toxicity in cell culture [186,192,217]. PGC-1α overexpression [52,212,218,219,220,221,222] or stabilization [211] can also prevent MPTP-mediated neurotoxicity in vivo. Recently, it has also been discovered that ferulic acid can reinstate mitochondrial dynamics through PGC-1α expression modulation in 6-hydroxydopamine lesioned rats [223].

Despite the fact that *TFAM* expression is not reduced in PD postmortem tissue [181], multiple lines of evidence suggest that dopaminergic neurons are vulnerable to disruptions in mitochondrial DNA (mtDNA) maintenance and/or regulation, potentially independent of PGC-1α. mtDNA maintenance depends on several nuclear encoded genes that form the replisome for mtDNA replication. These genes are mtDNA polymerase gamma 1 (*POLG1*), *TFAM* (discussed above), and the DNA helicase TWINKLE (*TWNK*). Mutations in these genes increase the risk of PD [224]. POLG1, like other polymerases, controls mtDNA synthesis, repair and replication [225]; the length of a CAG repeat in exon 2 directly correlates with function of the protein, and increased number of repeats has been associated with PD [226,227]. Also, mice expressing a proof-reading-deficient version of POLG1 on a PARKIN-deficient background exhibit an increase in pathogenic mtDNA mutations, motor deficits, and dopaminergic cell loss [228].

There have also been mutations identified that lead to reduction in mtDNA copy number in PD patients [229]. Accordingly, deletion of *Tfam* in dopaminergic neurons of mice (the Mito-Park model) causes a depletion of mtDNA and age-related loss of dopaminergic neurons of the SNc [230], and mice expressing mutant forms of *Twnk* selectively in dopaminergic neurons exhibit an increase in mtDNA mutations and dopaminergic neuron degeneration with a Parkinson’s-like motor phenotype [231]. Additionally, immunoprecipitation analyses have revealed that PARKIN itself can directly bind to mtDNA and TFAM, suggesting that PARKIN could directly affect the mitochondrial genome independent of PGC-1α [232,233,234].

Considering the relatively low basal expression of *Ppargc1a* and *Tfam* mRNA in dopaminergic neurons in mice (Figure 1a), it is possible that any stressors which reduce or impair the expression or function of these genes will overwhelm the ability of mitochondria to compensate, leading to cell loss. This relative deficiency in *Tfam* mRNA could also be a sign of reduced mitochondrial number in vivo, as has been reported in dopaminergic neurons versus non-dopaminergic neurons of the mouse SNc [235]. However, experiments with cultured SNc dopaminergic neurons actually suggest that SNc dopaminergic neurons have higher basal oxidative phosphorylation rates, higher mitochondrial density, and increased ROS production with respect to relatively resistant dopaminergic neurons derived from the ventral tegmental area [236]. Further experiments are required to resolve these findings; if dopaminergic neurons do, in fact, have reduced mitochondrial density as compared to other neurons, pharmacological strategies which are meant to target existing mitochondria may not be very effective in preventing dopaminergic cell loss.

### 6.3. Developmental Disorders

The observations of PGC-1α enrichment in cortical PV-IN populations and its requirement for the expression of developmentally regulated PV-IN-enriched genes raise questions about its potential involvement in developmental disorders. In fact, the chromosomal location including *PPARGC1A* has been linked to risk for schizophrenia and bipolar disorder [237,238,239,240], and a recent genome-wide association study found a significant association between schizophrenia and *rs215411*, a single-nucleotide polymorphism ~370 kb downstream of *PPARGC1A* [241,242].

A potential role for PV-IN dysfunction in schizophrenia pathophysiology was first suggested by a study indicating a reduction in *PVALB* mRNA expression in cortical interneurons without a reduction in PV-IN number [243]. Follow-up studies confirmed this reduction in *PVALB*, accompanied by reductions in *LHX6* [244], which encodes a transcription factor involved in PV-IN specification during development [245]. Subsequent studies demonstrated that the deletion of the obligatory subunit of the NMDA receptor, NR1, from PV-INs in mice can generate the behavioral characteristics of autism and schizophrenia, including abnormalities in sensorimotor gating, social behavior, and working memory [77,246,247,248,249,250], with enhanced cortical and hippocampal gamma rhythms at baseline [77,250,251]. Exposure of these mice to social isolation causes an age-related reduction in the expression of *Ppargc1a* mRNA and PGC-1α protein in the cortex of these mice, associated with an increase in oxidative stress markers in PV-INs [59].

With these studies in mind, we explored the expression of PGC-1α and PGC-1α-dependent genes in postmortem cortex of patients with schizophrenia [252]. We found that while *PPARGC1A* mRNA expression was not changed, mRNA for *PVALB*, *SYT2*, *NEFH*, and *CPLX1*, the PGC-1α-dependent transcripts identified in ([70]), were reduced. Interestingly, bioinformatics analysis of the promoters of these genes revealed enrichment of consensus binding sites for NRF-1, and *NRF1* mRNA expression was reduced in the cortex of these patients [252]. It’s possible that NRF-1 can mediate PGC-1α-dependent or independent regulation of neuron-enriched genes; while NRF-1 was initially identified as one of seven factors with consensus binding sites enriched in ubiquitously-expressed genes [253], multiple studies have shown that NRF-1 and/or NRF-2 are involved in the regulation of genes enriched in neurons such as neuronal nitric oxide synthase [254], AMPA receptor subunit 2 (*GRIA2*; [255,256]), NMDA receptor subunits NR1 (*GRIN1*) and NR2B (*GRIN2B*; [257]) and GABA receptor B1 (*GABRB1*; [258]). NRF-1 could serve as a critical transcription factor for the induction of cell-type-specific programs, depending on what combination of coactivators are present.

Along those lines, a disruption in transcriptional programs for PV-IN synaptic maturation and mitochondrial function has been found in postmortem tissue from patients with autism, bipolar disorder, and schizophrenia [259,260], with reduced abundance of mitochondrial electron transport chain protein complexes in both disorders [261,262]. A number of genes involved in mitochondrial function and responsive to PGC-1α overexpression in human neuroblastoma cells [70] are reduced, including *ATP5A1* [259,260], glutamic-oxaloacetic transaminase 1 (*GOT1*; [259]), *IDH3A* [260], and malate dehydrogenase 1 (*MDH1*; [260]). These findings are concordant with reports of mitochondrial dysfunction in autism (reviewed in [263] and [264]) and schizophrenia (reviewed in [265]). Transcriptional studies also reported decreased expression of *PVALB* [259,260], *SYT2*, *NEFH*, and glutamic acid decarboxylase 1 and 2 (*GAD1* and *GAD2*) [259], indicating either the loss of expression of these markers and mitochondrial genes in existing interneuron populations or the loss of these neurons.

A limitation of these studies is that the majority of transcriptional profiling was performed in brain homogenates, making it challenging, if not impossible, to determine whether mitochondrial transcripts are downregulated in specific cell types. Recently, a human pluripotent cell line protocol was developed to generate homogeneous cultures of cells with the properties of PV or somatostatin-expressing cortical interneurons which originate from the medial ganglionic eminence [266,267]. Using this protocol, Ni et al. 2020 [268] observed a reduction in the expression of genes involved in oxidative phosphorylation in iPSCs differentiated into interneurons but not pyramidal neurons from schizophrenia patients. These reductions were accompanied by decreased mitochondrial respiration and neurite arborization in interneuron cultures, which could be ameliorated by provision of a combination of alpha lipoic acid and acetyl-L-carnitine but not Coenzyme Q10 or N-acetyl cysteine. This study suggests that mitochondrial abnormalities in the cortex of patients with schizophrenia may arise from transcriptional alterations in interneurons; however, additional studies are needed to determine whether changes in transcription and function arise from disruption in nuclear or mitochondrial gene regulatory programs, as several of the reduced genes are expressed by the mitochondrial genome (ND2, ND5). Importantly, no statistically significant differences were noted in the percentage of neurons which express *GAD1*, indicating that interneuron-like cells derived from schizophrenia patients were differentiating normally in culture. The authors do not report whether the expression of PV was different in the cultures, which is an important consideration when trying to compare iPSC studies with in vivo findings.

Mitochondrial dysfunction and oxidative stress has been reported for PV-INs in a number of models of neurodevelopmental disorders, including 22q11.2, 15q13.3, or 1q21 microdeletions, fragile X syndrome, and various developmental models of schizophrenia (reviewed in [269]), suggesting that PV-IN impairment could be a common theme in neurodevelopmental disorders. Also, a recent article demonstrated that the deletion of COX10 from PV-expressing neurons in mice causes impairments in social behavior and sensori-motor gating, without affecting the total number of PV-INs in the cortex [270]. Increased gamma oscillatory power is also observed, consistent with models of NR1 deletion in PV-INs (above). Altogether, these findings suggest that disruption of PGC-1α and/or mitochondrial genes in PV-INs can cause alterations in inhibitory neurotransmission in numerous developmental disorders, making it important to develop strategies to rescue these pathways.

## 7. Viable Avenues for Drug Discovery and Therapeutic Intervention?

Strategies to improve mitochondrial function have been under consideration for the treatment of neurological and psychiatric disorders for quite some time, with limited success in clinical trials [271,272,273,274,275], despite promising results in animal models [276,277,278,279,280,281]. Even in individuals with genetic mutations that cause mitochondrial dysfunction and multi-organ disease, there are few treatment options [282]. PGC-1α was initially viewed as an ideal candidate to provide a full transcriptional program for enhancing citric acid cycle flux and oxidative phosphorylation capacity in parallel with the upregulation of genes involved in antioxidant defense. Here, we discuss commonly applied overexpression and pharmacological strategies for increasing PGC-1α expression or activity, highlighting potential adverse effects of excessive PGC-1α overexpression in vivo and the need to consider its cell-type-specific expression pattern when choosing approaches which target endogenously expressed PGC-1α.

### 7.1. Overexpression of PGC-1α

As mentioned above, the overexpression of PGC-1α can be neuroprotective in a number of mouse models, including HD, PD, and even kainic acid-induced status epilepticus [202]. This does not mean, however, that overexpression does not come without potentially unwanted side effects. The overexpression of PGC-1α can cause extreme mitochondrial biogenesis and hypertrophy in heart tissue [283], an uncoupling of mitochondrial respiration and reduction in ATP availability in skeletal muscle [284], and suppressed expression of transcription factors involved in the maintenance of phenotype and survival in dopaminergic neurons of the SNc, causing cell death [212,285]. While it is possible that the finer control of PGC-1α overexpression levels could mitigate some of these detrimental effects, it is still possible that PGC-1α overexpression, especially in cell types which do not normally express it, could interfere with normal transcriptional processes and/or cause the activation of transcriptional programs which negatively impact cell function.

### 7.2. Stimulating Increases in Endogenous Expression of PGC-1α and PGC-1α-responsive Genes

To avoid untoward effects of PGC-1α overexpression, it is possible to stimulate the modest upregulation of PGC-1α using a number of different strategies including exercise [84] or pharmacological agents, the majority of which are proposed to act by increasing AMPK activity and/or inhibiting SIRT1 activity [286,287]. A number of nutritional supplements such as quercetin [288], metformin [289,290], and the creatine analog beta guanidinoproprionic acid [120,291,292] can increase PGC-1α, NRF-1 and TFAM expression in the brain, potentially through the activation of AMPK, which can directly phosphorylate PGC-1α and increase its activity at its own promoter [293]. Activation of AMPK can also be stimulated by compounds such as 5′-aminoimidazole-4-carboxamide-1-beta-D-ribofuranoside (AICAR), leading to the attenuation of age-associated decline in mitochondrial biogenesis in skeletal muscle [294]; however, the effects on the brain are short-lived [295,296].

SIRT1, an NAD-dependent deacetylase, can deacetylate PGC-1α [297,298,299], causing a reduction in its activity. Thus, when SIRT1 was identified as a potential target for resveratrol, a compound enriched in red grapes [300], resveratrol became a popular strategy for the induction of endogenous PGC-1α activity and/or expression. While there are reports of resveratrol treatment increasing PGC-1α, NRF-1, and TFAM expression in the hippocampus [301] with some evidence for effects on cognition and mood in mouse models [302,303], trials in humans have been inconclusive [301], potentially due to its low bioavailability [304].

Here, it is important to point out that the majority of studies which measure the expression of PGC-1α and its dependent genes in the brain have not used techniques with cell-type-specific resolution nor have they measured the expression of neuron-enriched PGC-1α-responsive genes such as *Syt2*, *Cplx1*, and *Nefh*. Thus, in each tissue, the power to detect changes may be low (especially in the case of interneuron-expressed PGC-1α). Also, it is difficult to predict how any changes in PGC-1α will affect circuit function without knowing the neuroanatomical location of those changes. It is also important to consider the high concentration of PGC-1α in inhibitory neurons of the midbrain, including PV-expressing neurons of the substantia nigra pars reticulata. Changes in PGC-1α and its targets, when detected in homogenates of midbrain, could be reflecting this population instead of the dopaminergic neurons of the SNc.

**Figure 3:**
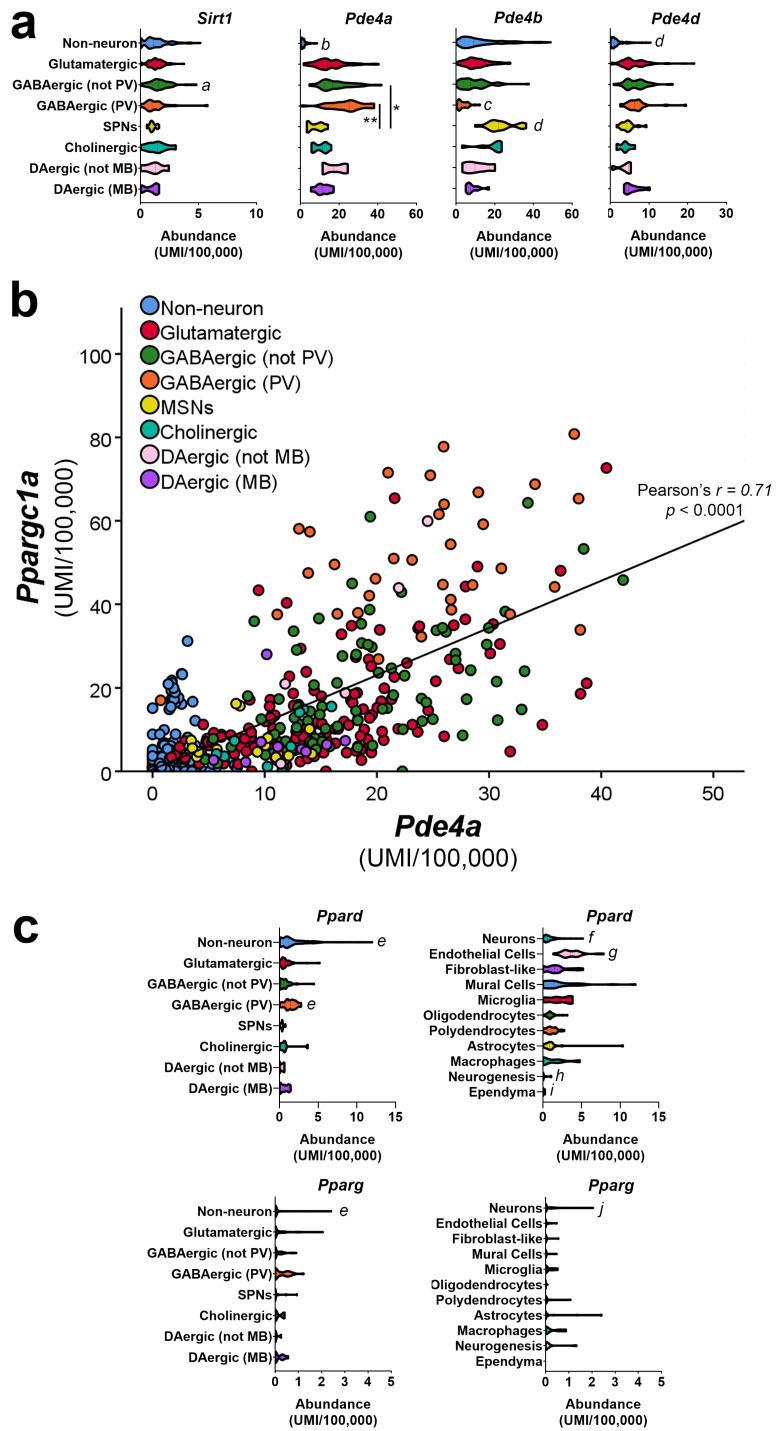
(**a**) A publicly available single-cell transcriptomic dataset [64] was used to explore cell-type-specific mRNA abundance of putative drug targets for manipulation of PGC-1α expression and/or activity. While *Sirt1* mRNA expression is low and ubiquitous, other transcripts which are putative targets of resveratrol are expressed more abundantly and in different cell-type-specific distributions. Note the difference in the x-axes scales. (**b**) A strong correlation was observed between the cell-type-specific abundance of *Ppargc1a* and *Pde4a* mRNA. (**c**) *Ppard* shows differential enrichment in neuronal and non-neuronal populations, while *Pparg* is expressed relatively lowly at the mRNA level. Note the differences in the x-axes scales. Kruskal-Wallis analyses were used in a and c, with * *p* < 0.05, ** *p* < 0.01, and letters indicating other statistically significant differences <0.05. *a* = different than non-neurons; *b* = different than all the rest; *c* = different than all but DAergic (not MB) and DAergic (MB); *d* = different than all but cholinergic and DAergic (not MB); *e* = different than glutamatergic, GABAergic (not PV), and SPNs; *f* = different than endothelial cells, fibroblast-like, and mural cells; *g* = different than all but microglia; *h* = different than all but macrophages and astrocytes; *i* = different than all but fibroblast-like, mural cells, microglia; *j* = different than fibroblast-like, mural cells, oligodendrocytes, and polydendrocytes; UMI = unique molecular identifier. PV = parvalbumin-expressing. SPNs = spiny projection neurons. MB = midbrain.

Furthermore, it is important to consider the colocalization of proteins which are proposed to act in concert to respond to the activation of AMPK and other upstream signaling cascades. Related to the potential actions of resveratrol in neurons, more recent studies suggest that phosphodiesterase IV (PDE4) is a putative target for resveratrol, with resveratrol-mediated PDE4 inhibition causing an increase in PKA activity, increased availability of cAMP, and increased PGC-1α activity/expression [305,306,307,308,309]. Interestingly, rolipram, a potent inhibitor of PDE4, shows antidepressant effects in humans [310] and neuroprotective efficacy in the quinolinic acid [311] and R6/2 models of HD [160,312]. However, due to gastrointestinal side effects and a narrow dosing window, it is not used clinically [310]. Regarding its potential cellular targets in vivo, analyses of the cell-type-specific distributions of different PDE4 isoforms in mouse single-cell transcriptomic data [64] indicate that *Pde4a*, *Pde4b*, and *Pde4d* are more abundant in all cell types than *Sirt1* and that *Pde4a* and *Pde4d* are expressed in PV-INs, similar to *Ppargc1a* (Figure 3a; *Pde4c* very low, not shown). *Pde4a*, in particular, shows a strong correlation with *Ppargc1a* across all cell types (Figure 3b). Further experiments could explore the involvement of specific PDE4 isoforms in gene regulation in PGC-1α-expressing cell types.

### 7.3. Specifying PGC-1α-responsive Pathways by Activating PGC-1α-interacting Transcription Factors

Another strategy for enhancing PGC-1α-dependent pathways is to use agonists for PGC-1α-interacting factors. The majority of these approaches focus on strategies for promoting the activity of members of the PPAR family with agonists, which have anti-diabetic properties. In the context of HD, activation of PPAR-dependent pathways may not only have the ability to increase PGC-1α-dependent gene expression but could prevent the interference of mtHTT with transcriptional programs. mtHTT can directly bind PPARδ to repress its transcriptional activity, which can be partially rescued by PGC-1αα in culture [108]. A loss of PPARδ contributes to several features of HD and pharmacological activation of PPARδ improves motor function and cell viability in vitro [108] and in vivo [313]. A subsequent study [314] demonstrated the neuroprotective efficacy of bexarotene, a small compound with RXR agonist activity; bexarotene reversed reductions in mtDNA content, genes involved in mitochondrial respiration, and mitochondrial structural abnormalities, accompanied by increased mtHTT clearance. The effects of bexarotene were dependent on PPAR, consistent with the evidence for the existence of RXR/PPARd heterodimers [315]. Similar effects, including the effects on PGC-1αα expression in the brain, have also been seen using rosiglitazone, a PPARγ agonist, or pan-PPAR activators in HD models [316,317,318,319]. Interestingly, *Ppard* transcript is more abundant in the mouse brain than *Pparg*, with enrichment of *Ppard* in cerebral endothelium and PV-expressing neurons (Figure 3c). It would be important to determine whether the effects of these agonists are due to enhancing transcription in restricted cellular populations.

### 7.4. Challenges with Using PGC-1α Expression or Mitochondrial Gene Expression as Readouts in Primary Screens of Cell Culture Systems

Drug development campaigns have been undertaken to identify novel PPAR agonists which increase PGC-1α activity; however, it is important to consider that undifferentiated cell systems, which are often used in primary screens for the identification of novel active compounds, could exhibit indirect induction of PGC-1α and genes involved in oxidative phosphorylation as part of the differentiation process. For example, PGC-1α can be upregulated by differentiation in SH-SY5Y neuroblastoma cells using histone deacetylase inhibitors [57]. While it would be attractive to use these data to propose HDAC inhibition as a strategy for driving PGC-1α-dependent gene programs, it is likely that PGC-1α upregulation is a consequence of cellular differentiation.

With the recent ability to derive cultures with neuronal properties from patient cells, many groups are using neurons derived from induced pluripotent stem cells (iPSCs) or directly induced from fibroblasts as platforms for drug discovery for neurological [320] and psychiatric diseases [321,322]. In light of the cellular localization of PGC-1α in the brain, it would be desirable to use cell types with high PGC-1α expression for drug development screens. However, it has been challenging to develop culture models of differentiated neurons with the phenotype of PV-expressing neurons, until recently (see [266,267,268]). While recent studies suggest that deriving neurons directly from fibroblasts without going through the process of iPSC reprogramming can avoid rejuvenation effects of the culture process [320,323], it is still important to consider that these cell culture systems may not fully reproduce the metabolic or mitochondrial state of differentiated cells in vivo [324,325], as neurons derived from iPSCs exhibit a higher glycolytic activity than mouse primary cultures [326]. However, some studies indicate that metabolic alterations due to disease state can still be maintained in neurons derived from patient iPSCs [268,327]. Even so, caution should be taken when using these types of cells to identify novel compounds which engage processes normally associated with differentiated cell types. Along those lines, primary screen approaches have recently started to steer away from traditional phenotypic readouts such as neurite outgrowth and luciferase reporter assay measures, recognizing that responses in cell lines may be cell-type- and context-specific [328]. Primary screen strategies which employ measures of direct protein-protein interactions as readouts, followed by a rigorous process of ruling out off-target effects, are more likely to successfully identify potent and selective compounds.

## 8. Conclusions

**Figure 4:**
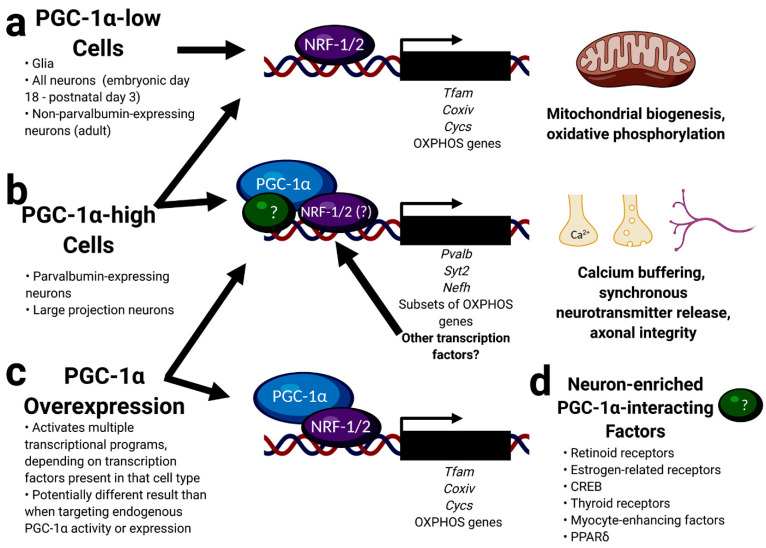
(**a**) In the brain, maintenance of NRF-1-target genes such as *Tfam* occurs independently of PGC-1α expression (no reduction in expression in brain of knockout mice [79]). (**b**) Brain maturation is associated with an increase in PGC-1α expression and the upregulation of genes enriched in parvalbumin-expressing neurons (*Pvalb*, *Syt2*, *Nefh*) as well as subsets of genes involved in oxidative phosphorylation. These genes could be driven by PGC-1α-containing complexes or by transcription factors regulated by PGC-1α. Any interference with PGC-1α expression and/or its interactions with transcription factors have the potential to impair the expression of synaptic genes and subsets of nuclear-encoded mitochondrial genes. These genes are reduced to the greatest extent in forebrain of PGC-1α knockout mice. (**c**) With overexpression, PGC-1α has the potential to engage transcriptional programs for mitochondrial respiration/biogenesis and neuronal transmission and integrity, depending on the cell type and transcription factors present. (**d**) Currently, it is not clear which transcription factors are required for the induction of neuron-enriched PGC-1α-dependent genes, although there are a number of possibilities. Created with BioRender.com.

Subtle changes in the expression of nuclear-encoded mitochondrial genes in the brain of various PGC-1α-deficient mouse models and differences in cell-type-specific expression patterns between PGC-1α and its interacting transcription factors raise questions about the extent to which PGC-1α deficiency causes neuronal dysfunction in neurodegeneration. However, modulation of the activity of PGC-1α and/or its interacting factors could still be a possible route for neuroprotection (see model of PGC-1α’s actions in neurons, Figure 4).

A substantial amount of evidence indicates that the overexpression of PGC-1α in different tissue types can drive the expression of nuclear-encoded genes involved in mitochondrial biogenesis, citric acid cycle flux, and oxidative phosphorylation. Concerns about neurotoxic side effects of PGC-1α overexpression highlight the need for targeting endogenous cellular pathways to enhance the expression of genes involved in mitochondrial function in disease states. When considering different strategies, multiple factors should be considered, including the cell-type-specific expression of PGC-1α and/or transcription factors involved in the regulation of mitochondrial genes, the cell-type-specific expression pattern of the intended target, and the experimental readouts used to confirm target engagement. Strategies promoting the coordinated upregulation of PGC-1α and PGC-1α-interacting factors involved in mitochondrial gene regulation may have the most promise.

## Data Availability

Raw data used for single-cell analyses are available at Dropviz.org [64].

## Supplementary Materials

The following are available online at https://www.mdpi.com/2073-4409/10/2/352/s1, Table S1: Summary of studies mentioned in the main text which reported the expression of PGC-1α transcript or protein in disease states.
