# Supplementary material for: Dysregulation of PGC-1α-Dependent Transcriptional Programs in Neurological and Developmental Disorders: Therapeutic Challenges and Opportunities
Source: Cells. 2021 Feb 9;10(2):352. doi: 10.3390/cells10020352 (PMC7915819; doi:10.3390/cells10020352)
Supplement: Supplementary file 1 [file cells-10-00352-s001.pdf]

Huntington Disease

Species	Tissue	PGC-1α Expression	Protein/Transcript	Downstream Genes	Expression	Citation	PMID
Human	Presymptomatic, postmortem caudate	Decreased	Transcript	Not measured	N/A	[112]	17018277
Human	Presymptomatic, postmortem cerebellum, hippocampus	No Change	Transcript	Not measured	N/A	[112]	17018277
Human	Presymptomatic, postmortem caudate (PMID16467349)	Decreased	Transcript	*ACADM, COX7C, CYCS, LDHB, NDUFS3, NDUFB5*	Decreased	[113]	17055784
Human	Brain lysates	Decreased; clinical grade dependent	Protein	*TFAM*	Decreased	[94]	20660112
Human	Symptomatic, putamen	FL-PGC-1α inconsistent	Protein	Not measured	N/A	[118]	21757867
Human	Symptomatic, putamen	NT-PGC-1α decr. grade 2, incr. grade 3&4	Protein	Not measured	N/A	[118]	21757867
Human	Myoblasts	FL-PGC-1α inconsistent	Protein	Not measured	N/A	[118]	21757867
Human	Myoblasts	NT-PGC-1α decreased	Protein	*NRF1, TFAM*	Decreased	[118]	21757867
Human	Symptomatic, muscle biopsy	Decreased	Transcript	*NRF1, TFAM*	No change	[115]	19460884
Mouse	STHdhQ111 (HD KI: PMID11092756)	Decreased	Protein/Transcript	*Coxiv, Cycs*	Decreased	[112]	17018277
Mouse	STHdhQ111	NT-PGC-1α decreased	Protein	Not measured	N/A	[118]	21757867
Mouse	STHdhQ111	Decreased	Transcript	*Cycs*	Decreased	[115]	20665636
Mouse	STHdhQ111	Decreased	Transcript	Not measured	N/A	[128]	30941017
Mouse	STHdhQ111	Decreased	Transcript	Not measured	N/A	[114]	22589249
Mouse	STHdhQ111	FL-PGC-1α inconsistent	Protein	Not measured	N/A	[118]	21757867
Mouse	R6/2; brain	FL-PGC-1α, NT-PGC-1α decreased	Transcript	*Tfam*	Decreased	[314]	22095692
Mouse	R6/2; muscle	FL-PGC-1α, NT-PGC-1α decreased	Transcript	*Tfam*	Decreased	[314]	22095692
Mouse	R6/2; brown adipose tissue	FL-PGC-1α, NT-PGC-1α decreased	Transcript	*Tfam*	Decreased	[314]	22095692
Mouse	R6/2, N171-82Q striatum (younger)	NT-PGC-1α decreased	Protein	Not measured	N/A	[118]	21757867
Mouse	R6/2, N171-82Q striatum (symptomatic)	NT-PGC-1α increased	Protein	Not measured	N/A	[118]	21757867
Mouse	R6/2, N171-82Q striatum	FL-PGC-1α inconsistent	Protein	Not measured	N/A	[118]	21757867
Mouse	R6/2; striatum	No Change	Transcript	*Pvalb*	Decreased; p>0.05	[45]	22916173
Mouse	LCM-captured medium spiny neurons (HD KI: PMID12926013)	Decreased	Transcript	Not measured	N/A	[112]	17018277
Mouse	LCM-captured nNOS interneurons (HD KI: PMID12926013)	Increased	Transcript	Not measured	N/A	[112]	17018277
Mouse	D9-N171-98Q striatum	Increased	Transcript	*Pvalb*	Increased	[105]	21177255
Mouse	N171-82Q striatum	Decreased	Transcript	*Cox6a, Cycs, Ldhb, Ndufs3, Tfam*	Decreased	[113]	17055784
Mouse	NLS-N171-82Q; soleus muscle	Decreased	Protein/Transcript	*Tfam*	Decreased	[115]	19460884
Mouse	NLS-N171-82Q; extensor digitorum longus	Decreased	Protein/Transcript	*Ppargc1b, Tfam*	Decreased	[115]	19460884
Mouse	NLS-N171-82Q;gastrocnemius	No Change	Protein/Transcript	*Tfam*	Decreased	[115]	19460884
Mouse	NLS-N171-82Q; striatum	Decreased	Protein/Transcript	*Cox2, Creb, Esrra, Nrf1, Nrf2, Ppard, Tfam*	Decreased	[116]	20529956
Mouse	NLS-N171-82Q; cortex	No Change	Protein/Transcript	*Coxiv, Esrra, Ppard*	Decreased	[116]	20529956
Mouse	NLS-N171-82Q; liver	Decreased	Transcript	*Hnf4a, Tfam*	Decreased	[116]	20529956
Mouse	NLS-N171-82Q; brown adipose tissue	Decreased;p >0.05	Transcript	*Ucp1*	Decreased; p>0.05	[116]	20529956
Mouse	HD knock-in series (PMID26908599); striatum	No Change	Transcript	*Ak1, Idh3a, Nceh1, Phyh, Wdr77*	Increased	[61]	29491012
Mouse	HD knock-in series (PMID26908599); cortex	No Change	Transcript	*Syt2, Nefh, Nceh1*, etc.	No change	[62]	32222555
Mouse	HD knock-in series (PMID26908599); hippocampus	No Change	Transcript	*Syt2, Nefh, Nceh1*, etc	No change	[62]	32222555
Mouse	PVcre-mthtt	No Change	Transcript	*Pvalb*	No change	[69]	24121117
Rat	Primary oligodendrocytes with lenti-mutant Htt exon1 (72Q)	Decreased	Transcript	*Hmgcs1, Hmgcr, Mbp*	Decreased	[116]	21715619
Rat	Primary neurons; full-length Htt (wtHtt with 15Q or mtHtt with 138Q)	No Change	Transcript	Not measured	N/A	[117]	19915593

Parkinson Disease

Species	Tissue	PGC-1α Expression	Protein/Transcript	Downstream Genes	Direction	Citation	PMID
Human	iPSC human derived	Decreased	Transcript	Not measured	N/A	[187]	24290359

Human	Substantia nigra from human post mortem PD patients Braak stage 5 or 6	Decreased	Transcript	Not measured	N/A	[188]	25363075
Human	Human H4 neuroglioma cells	Decreased	Transcript	Not measured	N/A	[188]	25363075
Human	Human derived iPSC dopaminergic neurons, LRRK2 mutation carriers	Decreased	Protein	Not measured	N/A	[197]	29129681
Human	Human substantia nigra samples from PD patients	Decreased	Protein/Transcript	Soluble Parkin decreased, Insoluble Parkin increased, NRF-1 decreased, PARIS increased	Decreased	[177]	21376232
Human	Human substantia nigra samples from PD patients	Decreased	Protein/Transcript	Insoluble Parkin, PARIS	Increased	[177]	21376232
Human	PARKIN deficient human dopaminergic neurons	Decreased	Protein/Transcript	PARIS/ZNF746	Increased	[181]	32795422
Human	PARKIN deficient human dopaminergic neurons	Decreased	Protein/Transcript	*PARK2*	Decreased	[181]	32795422
Human	Laser captured dopaminergic neurons, PD patients with Lewy pathology	No change	Protein/Transcript	Nuclear encoded mitochondrial genes	Increased/Decreased	[182]	20926834
Human	Human substantia nigra samples from PD patients	Decreased	Protein/Transcript	Not measured	N/A	[205]	30236862
Human	Human substantia nigra samples from PD patients	Decreased	Protein	*SDHA, TOMM20*	Decreased	[74]	27622213
Human	Neural progenitor Striatal cells	Increased	Transcript	*CAT1, CYCS, SOD1, SOD2, GPX1, UCP2*	Increased	[52]	17055439
Human	SHSY5's MPTP treated with Necdin lentiviral expression	Increased	Protein	Not measured	N/A	[207]	26971449
Mouse	A30P (Thy-1 alpha-syn mice) Brainstem and Cortex	Decreased	Transcript	Not measured	N/A	[188]	25363075
Mouse	Conditional Paris knockout mice injected with LentiGFP-Cre	Decreased	Protein/Transcript	*Paris/Zfp746*	Increased	[177]	21376232
Mouse	Conditional Paris knockout mice injected with LentiGFP-Cre	Decreased	Protein/Transcript	*Nrf1, Park2*	Decreased	[177]	21376232
Mouse	Conditional Pgc-1alpha knockout injected with AAV-CRE-GFP	Decreased	Protein/Transcript	*Sdha, Tomm20*	Decreased	[74]	27622213
Mouse	PGC-1alpha null mice	Decreased	Transcript	*Cytc, Ucp2, Ant1, Sod2, Gpx1, Cat1*	Decreased	[52]	17055439
Mouse	PGC-1alpha null mice	Decreased	Transcript	*Ucp3 , Sod1*	No change	[52]	17055439
Mouse	10T1/2 cells treated with hydrogen peroxide	Increased	Transcript	*Cytc, Ucp2, Ucp3, Ant1, Sod1, Sod2, Gpx1, Cat1*	No change	[52]	17055439
Mouse	PGC-1alpha null mice injected with MPTP	Decreased	Protein/Transcript	Not measured	N/A	[52]	17055439
Mouse	MPTP treated non-transgenic mice	Decreased	Protein/Transcript	*Esrra, Sirt3*	Decreased	[206]	26421366
Mouse	MPTP treated non-transgenic mice	Decreased	Protein/Transcript	*Atp5b, Sod2*	Increased	[206]	26421366
Mouse	MPTP treated non-transgenic mice with Sirt3 deletion in dopaminergic neurons	Decreased	Protein	*Esrra, Sirt3*	Decreased	[206]	26421366
Mouse	MPTP treated non-transgenic mice with Sirt3 deletion in dopaminergic neurons	Decreased	Protein	*Atp5b, Sod2*	Increased	[206]	26421366
Mouse	MPTP injected striatum, cortex,and cerebullum 90 minutes after injection	Increased	Transcript	Not measured	N/A	[209]	28161458
Mouse	MPTP injected striatum, cortex,and cerebullum 7 days after injection	No change	Transcript	Not measured	N/A	[209]	28161458
Mouse	MPTP injected striatum, cortex,and cerebullum 12 days after injection	No change	Transcript	Not measured	N/A	[209]	28161458
Mouse	MPTP-induced models of 6h-PSS, 24h-PSS, AdvPSS andESS of PD	Decreased	Transcript	*Kif1b, Mybb1a, Nrf1, Paris/Zfp746, Park2, Mybb1a*	Decreased	[210]	32280590
Drosophila	Drosophila S2 cell line or brain tissue with paris mutation	Decreased	Protein/Transcript	*Park2, Pink1*	Decreased	[179]	32138754
Rat	6-hydroxydopamie lesioned rats treated with ferulic acid	Increased	Protein/Transcript	*Drp1, Mfn2*	Increased	[219]	31657074

Neurodevelopmental Disorders

Species	Tissue	Direction	Protein/Transcript	Downstream Genes	Direction	Citation	PMID
Human	Human genomic DNA from major depressive disorder and controls	Increased	Transcript	Not measured	N/A	[236]	30381832
Human	Postmortem cingulate cortex tissue from patients with schizophrenia	No change	Transcript	*Syt2, Cplx1, Nefh, Parv, Nrf1*	Decreased	[248]	26683626
Mouse	Ppp1r2-Cre/fGluN1 knockout mice	Decreased	Protein	*Cat1, Gpx1, Sod1, Sod2*	Decreased	[53]	23348010

Supplemental Table S1. Summary of studies mentioned in the main text which reported the expression of PGC-1α transcript or protein in disease states.
